# Selection on ultrasonic call rate in neonatal rats affects low frequency, but not ultrasonic, vocalizations in adults

**DOI:** 10.1111/eth.13075

**Published:** 2020-06-23

**Authors:** Raffaela Lesch, Andrea Orozco, Margaret Shilling, Betty Zimmerberg, W. Tecumseh Fitch

**Affiliations:** 1 Department of Behavioral and Cognitive Biology, University of Vienna, Vienna, Austria; 2 Department of Psychology, Williams College, Williamstown, MA, USA

**Keywords:** development, domestication, human–animal interaction, play, pleiotropic effects, selection, ultrasonic vocalization, vocal communication

## Abstract

In this experiment, we studied a rodent model selected over 57 generations for high or low rates of ultrasonic vocalizations (USVs) during maternal separation as pups. We investigated the influence of this breeding on the adult animals’ subsequent vocal output, comparing acoustic variables across developmental stages. We hypothesized that selection on pup USV rate would impact adult USV production without affecting lower frequency calls. Contrary to this hypothesis, we found neither number of USV calls or other acoustic variables to differ among selected adult lines. Instead, we found that pup USV selection mainly affected adults’ low-frequency (human-audible) calls. Furthermore, low-frequency vocalizations did not fully fit a predicted correlation between body weight and fundamental frequency: high line males, although the heaviest on average, did not produce the lowest fundamental frequencies. Our findings suggest that selection for early ultrasonic vocal behaviour pleiotropically results in changes in anatomical production mechanisms and/or neural control affecting low-frequency calls.

## 1 Introduction

Pleiotropy is a pervasive phenomenon in evolution. Its relevance and existence were recognized early in medical syndromes, but the term “pleiotropy” was first coined by Ludwig Plate in 1910 (Stearns, 2010). Pleiotropy is an important concept with many different facets and definitions, but generally describes “the phenomenon of a single gene affecting multiple traits” (Paaby & Rockman, 2013). Here, we investigated the emergence of unselected by-products coupled to an originally selected for trait, including across developmental stages, as a possible example of pleiotropy, but we make no claims about genetics (Paaby & Rockman, 2013). Coherent development throughout developmental stages is essential for functionality in morphological structures (Cheverud, 1996), and pleiotropic traits can thus be representative of adaptive developmental processes that form and shape structures in organisms (Klingenberg, 2008).

A potential example of a pleiotropic effect can be found in dogs and other domesticated mammals. Human selection for tameness in these animals has been suggested to lead to downregulation of neural crest cell development and migration (Wilkins, Wrangham, & Fitch, 2014). Neural crest cells have multiple functions in embryonic development, generating pigmentation, sympathetic nervous tissues, craniofacial cartilages and bones, and interact in the formation of adrenal glands. A reduced functionality in neural crest cells is thus hypothesized to have direct effects on the animal’s arousal physiology (e.g. reduction of size and function of the adrenal glands and sympathetic nervous system), along with pleiotropic knockdown effects on pigmentation and skull shape. Changes in vocal behaviour are also known in domesticated animals, but we know very little about pleiotropic effects in the vocal or acoustic domain, due to a current lack of model organisms.

In the current study, we investigate potential pleiotropic effects of artificial selection on one vocal trait on other vocal traits across development. In particular, we examine how selective breeding on rat vocal output in an early developmental stage impacts vocal acoustics of later developmental stages.

Rats are an excellent model species to investigate pleiotropy in communication due to their rapid development and well-understood vocal repertoire. Rats produce a wide range of vocalizations and call types, and most of which are above the human hearing range, at over 20 kHz (Brudzynski, 2018). Calls above this threshold are called ultrasonic vocalizations (USVs). Young pups mostly produce USVs in the range of 30–65 kHz to maintain contact with their mother, who is crucial to their survival (Brunelli, Shair, & Hofer, 1994). Adult rats USVs split into two separate USV ranges to convey appetitive and aversive motivation. Appetitive calls centre around 50 kHz and aversive USVs at 22 kHz (Portfors, 2007). A large body of literature describes and uses these USVS as indicators of emotional states in rats (Brudzynski, 2015; Burgdorf et al., 2009; Simola & Brudzynski, 2018). However, both adults and pups also produce low-frequency calls (1–6 kHz) within the hearing range of humans. These calls serve to fend off predators and warn conspecifics, when the animal is in pain, and during rough-and-tumble play (Brudzynski, 2010, 2018).

The production mechanism of low-frequency calls is generally well understood in many mammals and results from vocal fold vibration in the larynx (Herbst et al., 2012) and is presumed to function similarly in rodents (Riede, York, Furst, Müller, & Seelecke, 2011; Roberts, 1975), and the mechanism of rodent USV production is debated. There is wide agreement that USVs are the product of a whistle mechanism (Johnson, Ciucci, Russell, Hammer, & Connor, 2010; Mahrt, Agarwal, Perkel, Portfors, & Elemans, 2016; Riede, Borgard, & Pasch, 2017; Roberts, 1975; Sanders, Weisz, Yang, Fung, & Amirali, 2001), but disagreement exists concerning the exact underlying production mechanism. Mahrt et al. (2016) suggests an intra-laryngeal planar impinging jet (Mahrt et al., 2016) to be responsible for USV production, while Riede et al. (2017) describe an edge-tone whistle mechanism as the source (Riede et al., 2017).

The present study investigates a selected line of rats, whose breeding was based on the production of infant USVs during maternal separation. This selection has resulted in two unique, stable lines, where pups emit USVs at high and low rates (Brunelli & Hofer, 2007). Infantile USVs are regarded as an indicator of the negative affective state of the pup (hence the term “distress calls”; Allin & Banks, 1972; Shair, 2007) since they decrease after the administration of anti-anxiety agents such as the benzodiazepines and neurosteroids (Carden & Hofer, 1990; Winslow & Insel, 1991; Zimmerberg, Brunelli, & Hofer, 1994). In earlier work, variations in acoustical features other than call rate were also found to be by-products of the selective breeding on these two lines. These unselected, pleiotropic by-products include longer call duration, louder relative amplitude and broader frequency bandwidth in the high-production line (Spence, Aslam, Hofer, Brunelli, & Shair, 2016). Studies of the behaviour of these high and low lines at later ages have documented the concurrent divergence of the lines into two “affective temperaments”. High line rats tend to be more anxious, depressed and less playful, with sympathetic nervous system over-activity, while low line rats tend to be more active and aggressive, with parasympathetic nervous system underactivity (Brunelli, 2005; Brunelli, Zimmerberg, & Hofer, 2010; Zimmerberg, Brunelli, Fluty, & Frye, 2005). Low line adult rats also perform better in spatial learning and object recognition tasks as compared to high line rats.

Burgdorf et al. (2009) independently produced two similar rat lines based on a different selection process. Their selection pressure was based on adult 50kHz USV responsiveness to positive social interaction (“tickling”). Adults of the responsive line produced a higher number of 50kHz calls as adolescents and adults, compared with the less responsive line. This selection for tickling responsiveness to positive social interaction not only led to the formation of two distinct lines, but it also led to the emergence of pleiotropic affective traits. Responsive lines with a high rate of 50kHz calls proved to be more stress resilient and had a more positive affectivity, compared with rats of the unresponsive line.

Both Brunelli-Hofer’s and Burgdorf’s rat lines have provided insight into pleiotropic changes affecting behaviour and physiology. To date however, we know little about how selection for vocal output at one stage might affect the vocal communication output at a different developmental stage. Our study bridges this gap by investigating the pleiotropic changes early selection pressure has on acoustic output later in development.

Based on the results presented by Burgdorf et al. (2009), and the selection pressure our rat lines were subjected to (i.e. to produce more pup USVs in the high line), we hypothesized that our adult rats would produce USVs in accordance with their behaviour as pups. One consideration supporting this hypothesis concerns the costliness of USV production: Compared with low-frequency squeaks which are very energy efficient, USV production has a higher metabolic cost (Kelm-Nelson, Lenell, Johnson, & Ciucci, 2018). Therefore, we hypothesized that selection pressure for increased pup USV production would also select for ability to sustain a higher level of communicative effort in adults. Second, in an earlier study, pup line USVs were reported to differ in call duration and range (Spence et al., 2016); our second hypothesis predicts that this will remain true for the adult rat line USVs. Finally, selection for USV rate in pups potentially influences the development and structure of the larynx, which in turn could impact the production of other calls. However, due to their different production mechanisms, low-frequency (human-audible) vocalizations produced by vocal fold vibration may be unaffected relative to high frequency whistles. Our third hypothesis thus predicts that USV-focussed selection will have little measurable impact on the low-frequency (audible) calls between lines.

## 2 Methods

### 2.1 Ethical approvals

All housing and testing procedures were approved by the Williams College Animal Use and Care Committee (ethical approval number ZB-B-17).

### 2.2 Line breeding

Subjects for this experiment were offspring from the 57th (adults) and 58th generation (pups) of N:NIH rats that were selectively bred for high and low ultrasonic vocalization rates as induced by brief (20 min) maternal separation at 10 days of age, with selection methods described in detail in Brunelli, Vinocur, Soo-Hoo, and Hofer (1997).

### 2.3 Housing

Mating was conducted within each line. Pregnant females, determined by the presence of a vaginal plug, were individually housed in plastic cages (45 × 25 × 15 cm) in an isolated nursery and had continuous access to standard laboratory chow and water throughout their pregnancies. The day of birth was denoted post-natal day (PN) 0. Distinct litters were used for pup and adult tests. Each litter was represented by no more than one male or one female in any behavioural test. For adult subjects, the pups stayed in the cage with their mother until weaning at PN 25. At weaning, the animals were housed in same sex pairs until 3 days prior to the start of habituation, when they were housed individually.

All rats were housed in standard rodent hanging cages and were given access to food and water ad libitum. All animals were kept on a 12/12 cycle of dark and light with the lights turning on at 06:00. Colony temperatures were maintained between 21.7 and 22.8°C with humidity levels between 44% and 55%. Both pups and adult rats were weighed after testing with a standard laboratory scale in grams with a two decimal place accuracy.

### 2.4 Pup test paradigm

**Figure 1:**
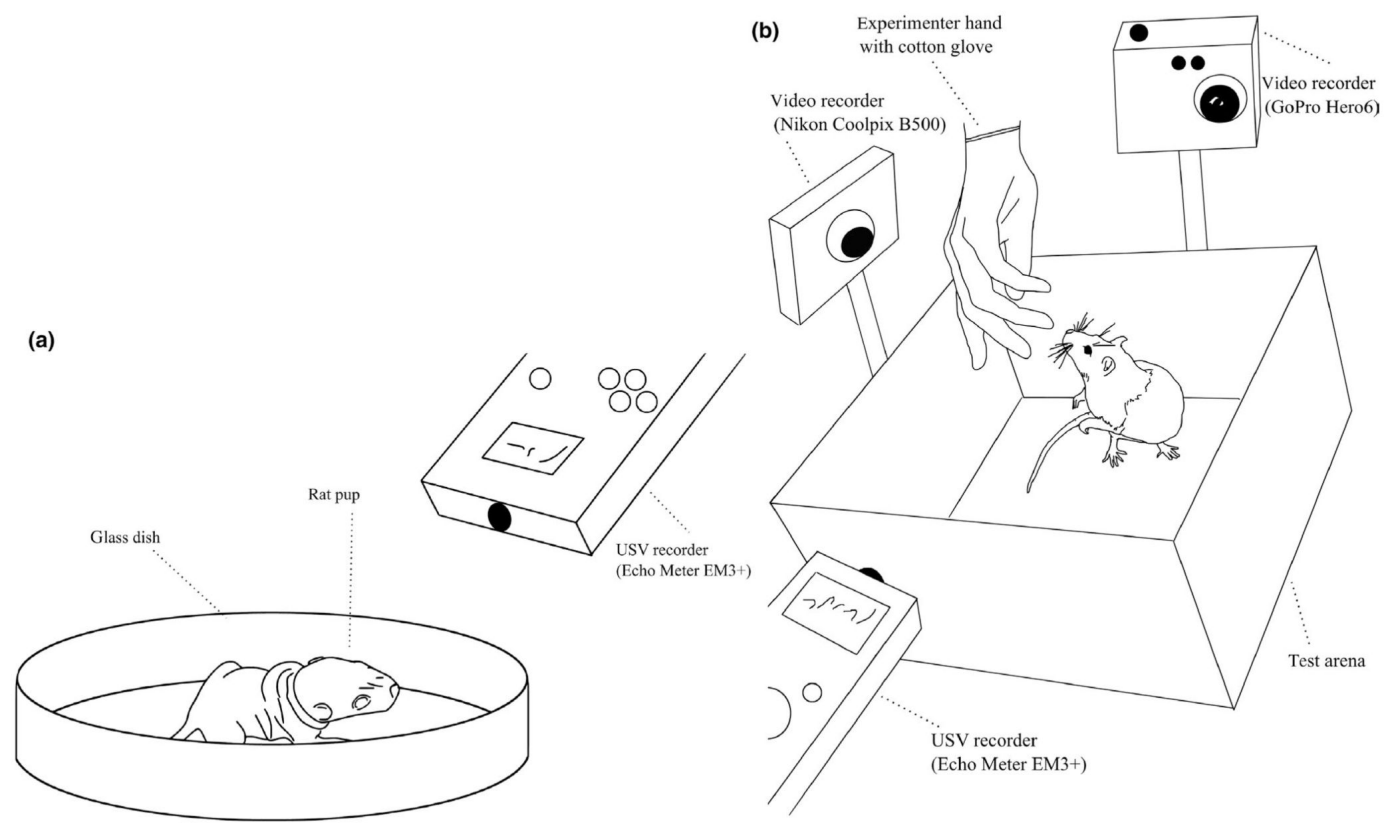
Schematic depiction of the two-minute recording period test set-ups for the elicitation paradigms. (a) Test set-up for the pup separation paradigm. The rat pup is isolated on a glass dish and recorded with a USV recorder. (b) Test set-up for the adult rat elicitation (“tickle”) paradigm. The experimenter is wearing a cotton glove and interacts with the rat in the test cage. The whole procedure is recorded with two video cameras and one USV audio recorder

Pup ultrasonic vocalizations were measured following the brief maternal separation paradigm (Brunelli et al., 1997). On PN day 10, a total of 50 individuals with a sex:line ratio of 11 high line males, 14 low line males, 12 high line females and 13 low line females were tested in this experiment. Each litter was represented by at most only one male and female pup from a total of 12 high line litters and 13 low line litters. Each litter was removed from the home cage by hand and placed in a transport cage with bedding. The transport cage was then taken to the dimly lit adjacent test room. The cage was placed on a heating pad set at medium (slightly below body temperature) for 20 min. After this maternal separation period, we randomly selected one male and one female subject for testing. Subjects were placed individually in a circular glass dish (10 cm tall and 20 cm in diameter) and taken to an adjacent testing room. We recorded ultrasonic vocalizations (USVs) for 2 min with an ultrasonic recording system Model 3EM+ from Wildlife Acoustics (https://www.wildlifeacoustics.com/) held approximately 20 cm over the container (Figure 1a).

### 2.5 Adult test paradigm

Adult rat vocalizations of both lines were measured using a tickling paradigm designed to elicit play behaviour and affiliative vocalizations in rats. This paradigm was developed by Panksepp and Burgdorf (2000) and since then has been adopted by other groups (LaFollette, O’Haire, Cloutier, Blankenberger, & Gaskill, 2017). We tested 51 adult rats recording 14 high line males, 13 low line males, 12 high line females and 12 low line females (not the same individuals tested as pups). One low line male rat was excluded on the first day of handling because it refused to interact with the experimenters. For the habituation and the experiment, the 50 remaining individuals were randomly split into three groups (Ns = 17, 17, 16). We staggered the starting day of each group in one day intervals and habituated and tested each group over four consecutive days. The whole adult elicitation (“tickle”) experiment and habituation procedure were completed over six consecutive days.

#### 2.5.1 Handling during habituation and testing

For the first two days of habituation, the rats’ cages were taken out of the holding shelf and placed on a cart. On the cart, the rats were carefully removed from their home cage by picking them up with one hand placed under the thorax and supporting them with the second hand under the hind legs. For habituation, they were placed in and habituated to a test cage (30 × 30 × 15 cm) for a duration of two minutes. After habituation, the rat was carefully picked up again and placed back into its home cage, which was returned to the holding shelf. Experimenters always wore the same reusable cotton gloves while handling the animals to ensure the same smell and texture for the rats. This same protocol was used on the third day of habituation and the testing day with the addition that the test cage was covered with a metal mesh to transfer the animal to the testing room. After removing the metal lid the animals were habituated/tested in the testing room for two minutes. After habituation/testing, the rat was carefully carried back in the secured test cage and placed back into its home cage.

#### 2.5.2 Habituation protocol

Following Panksepp and Burgdorf (Panksepp & Burgdorf, 2000), we adapted the habituation process as follows. Each day the habituation lasted two minutes.

Day 1: The experimenters slowly introduced each rat to human contact and interaction. We encouraged the rats to sniff the experimenter’s hand, and once the rat was comfortable with the experimenter, we softly stroked the animal’s head and back. The habituation was completed in a test cage in the same room in which the animals were housed.

Day 2: We introduced the rat to more contact with the human hand and tried to elicit a play response. Once they were comfortable with the protocol, we picked the rats up and tickled them on the belly. The habituation was again performed in a test cage in the housing room.

Day 3: We invited the rat to play with the human’s hand. All of the rats were comfortable being touched by the human experimenter within the first minute. Some rats started to chase the experimenter’s hand as a playful response. In the second minute, we picked up the rats and tickled their belly. The habituation was done in a test cage in the testing room.

#### 2.5.3 Test protocol

The test protocol started with tickling the rat on the back, neck, and head region. These short interactions were interspersed with play invitations to chase the experimenter’s hand (Figure 1b). The tickling and hand chasing alternated for the first minute. In the second minute, we picked up the rat and tickled it on the belly. This again was alternated with periods of inviting the rat to chase the experimenter’s hand. The whole test was recorded with the recording equipment specified below. We cleaned the plastic cages with the odour eliminating animal-safe cleaner “Odor Mute” (Hueter Toledo Inc, Bellevue, Ohio) prior to testing each individual.

### 2.6 Recording and analysis

#### 2.6.1 Recording equipment

We video recorded the affiliative/play task from two different angles using a Nikon Coolpix B500 (HD 1,920 × 1,080, mp4 format) and a GoPro Hero6 (HD 1,920 × 1,080, mp4 format). We audio recorded both the adult elicitation and pup separation experiment with a handheld bat detector (Echo Meter EM3+ from Wildlife Acoustics; https://www.wildlifeacoustics.com/). The sampling rate was set to 256 kHz for the tickle paradigm and 384 kHz for the separation task. The recordings were saved in wav format with 16-bit quantization. The audio trigger mode (real time expansion) was set to 18 kHz, 0 dB SPL and 3.0 s to record continuously. The audio recordings gave us a total of 102 min of adult and 100 min of pup recordings.

#### 2.6.2 Acoustic definitions

In this study, we defined calls based on physical frequency range. Below, we present commonly used definitions for USVs and audible vocalizations:

“Audible vocalizations”: Vocalizations with a fundamental frequency below 20 kHz and within the typical human hearing range.

“USVs”: Vocalizations with a fundamental frequency above the human hearing range; in rats vocalizations ranging from 20 to 80 kHz.

#### 2.6.3 Acoustic analysis

We performed the acoustic analysis with four custom semi-automated scripts. All pup and adult vocalization recordings were trimmed to the two-minute test length (using Praat (Version 6.0.23; www.fon.hum.uva.nl/praat/)) prior to running the scripts.

### 2.7 Adult analysis

The varying signal to noise ratio and diverse background noises (e.g. rat nails on the arena floor, sniffing the microphone, rustling of the fur) required hand labelling of all USVs and audible calls. After labelling an automated script exported the calls into individual wav files. In preparation for further analysis, all calls of most individuals were merged into two recoverable wav chains (audible and USV), but to avoid errors we created a separate USV chain for individual 38 due to his high number of 22-kHz calls. All chains were then processed by a semi-automated pitch tracking script that provided a manually adjustable visual representation of the proposed pitch tracking. For USV calls, the Praat internal function for the autocorrelation algorithm was set to: Advanced pitch settings: 0, 130000, “no,” 10, 0.0003, 0.00045, 0.3, 0.35, 0.002 with Pitch settings: 25000, 90000, “Hertz,” “autocorrelation,” and “automatic,” The same functions with the following setting changes were used for the audible vocalization chain: Advanced pitch settings: 0, 9000, “no,” 15, 0.03, 0.45, 0.01, 0.35, 0.14 and Pitch settings: 1400, 6000, “Hertz,” “autocorrelation,” “automatic.” Then, the pitch contour was extracted automatically with the Praat internal command “Extract visible pitch contour” for every call and saved as a pitch contour. A final automated script measured the acoustic parameters duration, $\bar{x}$ mean f0, max f0 and min f0 using the following Praat internal commands: Get total duration, Get mean(0, 0, “Hertz”), Get maximum (0, 0, “Hertz,” “Parabolic”) and Get minimum (0, 0, “Hertz,” “Parabolic”), respectively. These values were saved to a text file. F0 range was then calculated by subtracting each minimum f0 from the maximum f0.

### 2.8 Pup analysis

Because pups were generally immobile, there was less extraneous noise in their recordings, and this more consistent signal to noise ratio allowed us to use a completely automated system for the labelling and extraction of USV calls. We used the algorithm USVSEG (usvseg08r6; version 8, revision 6; 9.12.2018) by Tachibana, Kanno, Okabe, Kobayasi, and Okanoya (2020; running in MATLAB (version: R2018a)) for both labelling and the extraction. The USVSEG settings were set at time step (ms): 0.5, freq min (kHz): 20, freq max (kHz): 120, threshold (*SD*): 4.5, dur min (ms): 5, dur max (ms): 300, gap min (ms): 30, margin (ms): 15, wavfile output: on, image output: on, image type: orig, trace output: off, read size (s): 130 and map (kHz): 1,6. The algorithm saved all calls as individual wav files. They were manually concatenated into recoverable chains of calls for each individual using Praat. Just as with the adult analysis, all chains were put through a semi-automated script that tracked and extracted the pitch contour. The overall pitch tracking settings were pre-set (Advanced pitch settings: 0, 130000, “no,” 10, 0.0003, 0.00045, 0.35, 0.4, 0.002; Pitch settings: 30000, 73000, “Hertz,” “autocorrelation,” “automatic”) and could be adjusted and changed manually before the script ran through all calls and extracted each individual contour with the Praat internal command “Extract visible pitch contour.” The last fully automated script took acoustic measurements from the previously extracted pitch contours. Duration, minimum, maximum and mean fundamental frequency were extracted with the same Praat commands as before and f0 range was again calculated by subtracting each minimum f0 from the maximum f0.

### 2.9 Statistical analysis

Statistical analyses were performed in R (Version 3.6.1; www.r-project.org/) and RStudio (Version 1.2.1335; www.rstudio.com/) with the packages nlme, lme4, MASS, car, ggplot2, ggthemes, cowplot, multcomp, MuMIn, piecewiseSEM, r2glmm, visreg, dplyr, doBy, data. table, xlsx, GGally, openxlsx, car, readxl, plyr, stats, mclust, doBy, cowplot, gridExtra, gtable, grid, influence.ME, factoextra, olsrr and parallel.

We analysed calls collected from an N of 50 pups and 50 adults. We first used the package mclust to cluster all adult rat vocalizations and assign them to three categories: audible calls (<20 kHz), 22 kHz calls and 50 kHz calls (Portfors, 2007). The cluster analysis revealed seven categories of which four were in the 50 kHz range, two in the audible range and another one the 22 kHz call range. The four 50 kHz clusters were assigned to the 50 kHz category and the two audible call clusters to the audible call category. The 22 kHz range was only represented by 18 calls produced by one individual and therefore excluded from a separate analysis due to the small sample size. The 92 audible calls were analysed separately from the 390 50 kHz USVs. The different call contribution was taken into account for both the audible and 50 kHz data. This was done by including the individuals ID as a random effect in all models; allowing us to control for individuals of both lines contributing varying numbers of calls. Clustering prior to statistical analysis was not necessary for the 7,895 pup USV calls since pups only produce one range of pup specific USV calls.

We used generalized linear models (GLMs) of the lme4 package to fit all measured f_0_ parameters mean, maximum, minimum, range and duration, for all 3 categories: pup USVs, adult rat USVs and adult rat audible calls. For the pup data, no good model fit could be achieved while running GLMs on the complete data set due to singularity issues. Therefore, we collapsed all measurements down to the mean of the individual to achieve a distribution that could be analysed in our models. We then ran linear models for all acoustic measurements of pup USVs except for maximum f0 which required a generalized linear model with Gamma (log link) distribution. For adult rats, audible calls were analysed with linear mixed models, and for adult USVs we used generalized linear mixed models with Gamma (log link) distributions, but duration was fitted with a Gaussian (log link) distribution. We applied random effect intercepts for each individual in all adult rat models to correct for individual variation and call contribution. We had to refrain from creating random slopes for the individuals to avoid convergence issues. We tested all models for their stability, checked for collinearity issues and visualized their residuals to check for possible violations of distribution assumptions. Due to the sexual dimorphism in adult rats, we included a dummy-coded factor of rat line and sex resulting in four categories (high line male, low line male, high line female, low line female), dubbed line combinations. This line combination, bodyweight and their interaction were input as fixed factors to the full models and compared with null models lacking these fixed factors. To disentangle the involvement of sex and line, a third model including sex, bodyweight and their interaction were run and included as an additional control in the comparison of full-null model testing. The factors bodyweight, sex and line combination indicated possible collinearity issues with a VIF (variance inflation factor) value between 3 and 4. We plotted the affected factors and upon visual inspection we could confirm that there was considerable variation among these factors, and therefore, the VIF value was no problem for the model fit (Mundry, 2014).

## 3 Results

As described under “Statistical Analysis,” we created a dummy-coded factor for the combination of sex and line. For concision this factor will be referred to as “line combination” below.

### 3.1 Number of vocalizations in rat pups and adults

**Figure 2:**
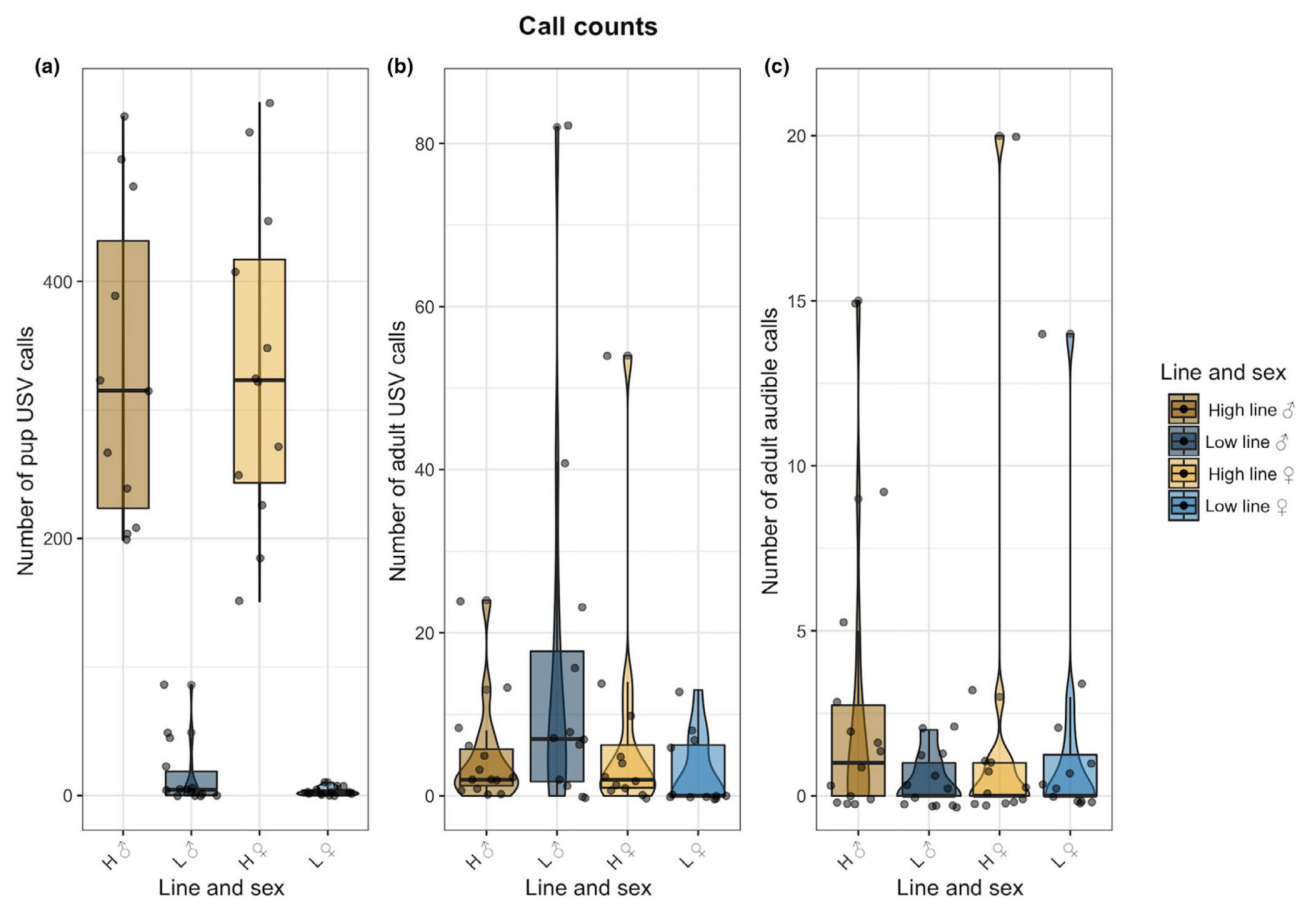
Total counts of pup and adult vocalizations per individual. The different lines are indicated by letters (H = high line, L = low line), and sex is indicated by symbols (♂ = male, ♀ = female). (a) Total number of USVs produced by pups plotted over the line and sex combinations. (b) Total number of USVs produced by adult rats and plotted over the line ad sex combinations. (c) Total number of audible calls produced by adult rats plotted over the line and sex combinations [Colour figure can be viewed at wileyonlinelibrary.com]

For the maternal separation paradigm, we found the full model for the number of rat pup calls to be significantly better than the null model (Pr(>Chi) < 2.2e-16). High line pups produced calls numbering in the hundreds, whereas low line pups barely produced any calls and never exceeded one hundred calls (Figure 2a; $\bar{x}\pm SD:\;\text{high}\;$ ♂: 331 ± 123, low ♂: 16 ± 26, high ♀: 332 ± 125, low ♀: 3 ± 3). Thus, the two lines show a distinct difference in the selected trait.

In contrast, in adults we did not find such a distinct difference in the number of calls, either for adult rat USVs or adult audible calls. The full model for adult USVs, including line combination, bodyweight and their interaction, was significantly better than the null model and the reduced model (Pr(>Chi) = 1.176e-10) in explaining our data’s distribution (Figure 2b; $\bar{x}\pm SD:\;\text{high}\;$ ♂: 5 ± 7, low ♂: 16 ± 24, high ♀: 8 ± 15, low ♀: 3 ± 5). The full model for adult audible calls was not significantly better than the reduced model just including sex, bodyweight and their interaction (Figure 2c; Pr(>Chi) = 0.372; $\bar{x}\pm SD:\;\text{high}\;$ ♂: 3 ± 4, low ♂: 1 ± 1, high ♀: 2 ± 6, low ♀: 2 ± 4). Thus, while pup USVs were much more frequent in the line selected for high vocal production, adult lines did not show such a clear distinction in the number of calls produced. Whereas the number of the adult audible calls did not differ across lines the significant difference found in the adult USV calls seems to be driven by the high number of USVs produced by low line males.

#### 2.3.2 Pup USV acoustics

**Figure 3:**
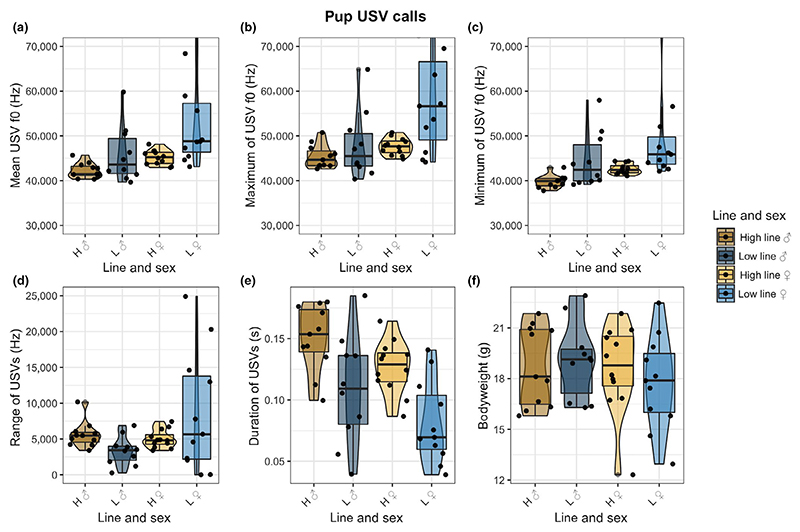
Pup USV acoustic measurements. The different lines are indicated by letters (H = high line, L = low line) and sex is indicated by symbols (♂ = male, ♀ = female). (a) Mean f0 of pup USVs plotted over the line and sex combinations. (b) Maximum f0 of pup USVs plotted over the line and sex combinations. (c) Minimum f0 of pup USVs plotted over the line and sex combinations. (d) f0 range of pup USVs plotted over the line and sex combinations. (e) Call duration of pup USVs over the line and sex combination. (f) Bodyweight in grams over the line and sex combination [Colour figure can be viewed at wileyonlinelibrary.com]

Pup USV measurements mean f0, maximum f0, minimum f0 and duration, recorded during the separation paradigm, were most accurately predicted by the full models including fixed factors line combination, bodyweight and their interaction (mean: Pr(>Chi) = 0.0165, max: Pr(>Chi) = 0.004972, min: Pr(>Chi) = 0.0124, dur: Pr(>Chi) < 0.000025). Pups at the age of testing did not differ significantly in bodyweight (Figure 3f; Pr(>Chi) = 0.6502). In both the mean f0 ($\bar{x}\pm SD:\;\text{high}\;$ ♂: 42,250 ± 1,670, low ♂:45,870 ± 6,302, high ♀: 45,181 ± 1,623, low ♀: 52,965 ± 9,861), maximum f0 ($\bar{x}\pm SD:\;\text{high}\;$ ♂: 45,381 ± 2,612, low ♂:47,951 ± 7,491, high ♀: 47,622 ± 1,877, low ♀:57,513 ± 10,715) and minimum f0 ($\bar{x}\pm SD:\;\text{high}\;$ ♂: 39,785 ± 1,407, low ♂: 44,603 ± 6,257, high ♀: 42,615 ± 1,172, low ♀:48,856 ± 8,995), high line males on average produced the lowest frequencies and low line females the highest (Figure 3a,b,c). Based on visual inspection, across these three f0 measures low line female and male pups produced USVs with a higher degree of variability in comparison with their high line counterparts. Average USV call duration was longest in males of the high line and lowest in females of the low line (Figure 3e; $\bar{x}\pm SD:\;\text{high}\;$ ♂: 0.15 ± 0.03, low ♂:0.11 ± 0.04, high ♀:0.13 ± 0.02, low ♀:0.08 ± 0.03). High line individuals of both sexes produced, on average, longer calls than low line pups. We did not find the f0 range model to be significantly better than the null model (Figure 3d).

### 3.3 Adult USV acoustics

**Figure 4:**
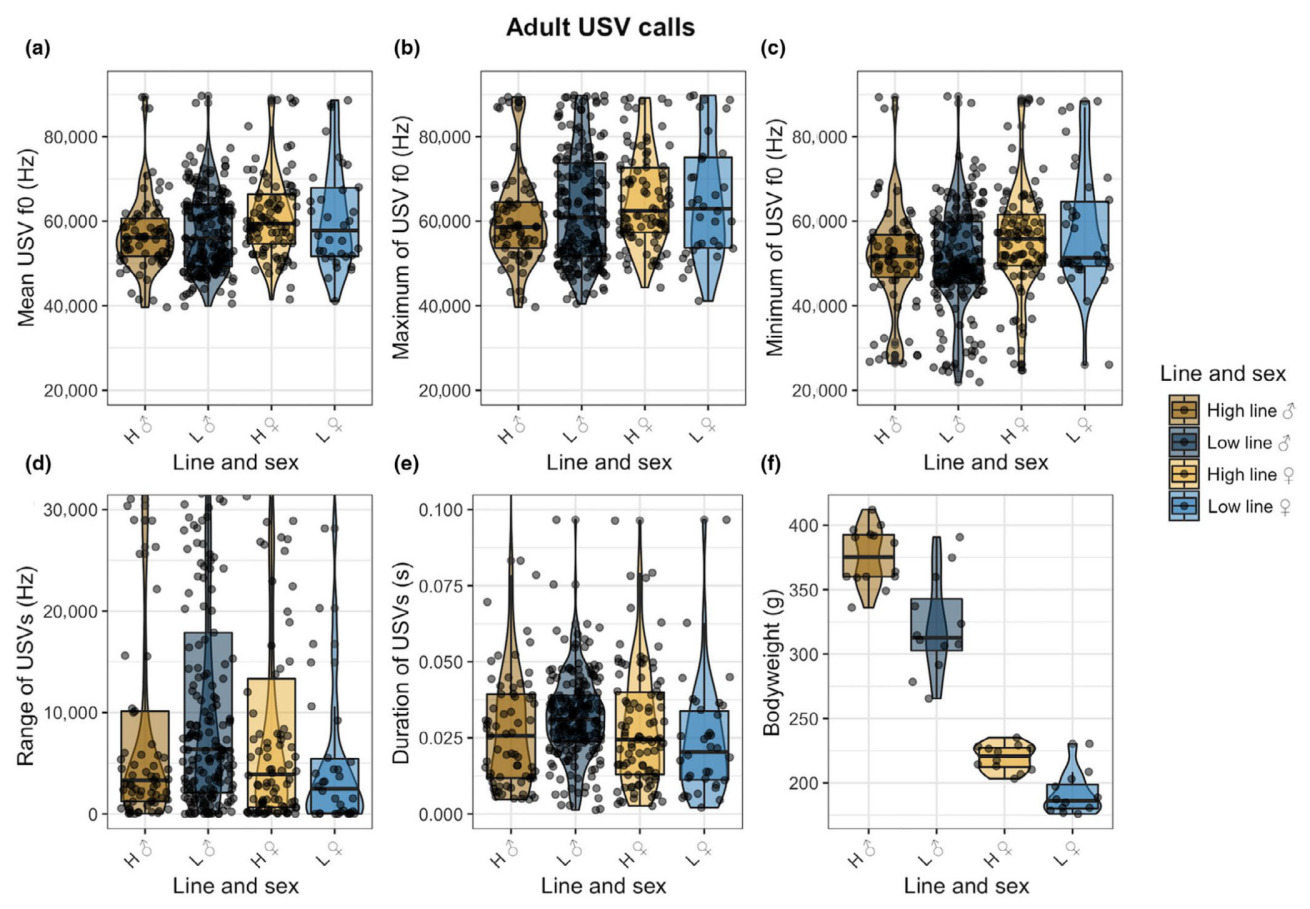
Adult USV acoustic measurements. The different lines are indicated by letters (H = high line, L = low line), and sex is indicated by symbols (♂ = male, ♀ = female). (a) Mean f0 of adult USVs plotted over the line and sex combinations. (b) Maximum f0 of adult USVs plotted over the line and sex combinations. (c) Minimum f0 of adult USVs plotted over the line and sex combinations. (d) f0 range of adult USVs plotted over the line and sex combinations. (e) Call duration of adult USVs over the line and sex combination. (f) Bodyweight in grams over the line and sex combination [Colour figure can be viewed at wileyonlinelibrary.com]

In contrast to the pup results, adult rat USVs did not differ across line combinations; mean f0 ($\bar{x}\pm SD:\;\text{high}\;$ ♂: 56,696 ± 8,690, low ♂: 57,381 ± 9,435, high ♀: 61,122 ± 9,741, low ♀: 61,096 ± 12,487), maximum f0 ($\bar{x}\pm SD:\;\text{high}\;$ ♂: 60,515 ± 11,234, low ♂:63,007 ± 13,271, high ♀:65,121 ± 11,059, low ♀:65,170 ± 14,205), minimum f0 ($\bar{x}\pm SD:\;\text{high}\;$ ♂: 51,173 ± 12,258, low ♂: 50,917 ± 11,930, high ♀: 55,512 ± 12,764, low ♀: 57,915 ± 14,115) and USV duration ($\bar{x}\pm SD:\;\text{high}\;$ ♂: 0.03 ± 0.04, low ♂: 0.03 ± 0.01, high ♀: 0.03 ± 0.02, low ♀: 0.02 ± 0.02). For all acoustic parameters, full generalized linear mixed models were not significantly better than the null model (Figure 4). Due to persistent overdispersion in the f0 range (1.6), the technically significant difference of Pr(>Chisq) = 0.0195 over the null model cannot be interpreted as reliable. Only the full model for bodyweight was significantly better than the null model, showing a distinct difference in bodyweight explainable by both sex dimorphism and line combination (Pr(>Chisq) < 2.2e-16). Thus, selection focused on pup USVs did not have a corresponding effect on adult USV acoustics.

### 3.4 Adult audible (low-frequency) acoustics

**Figure 5:**
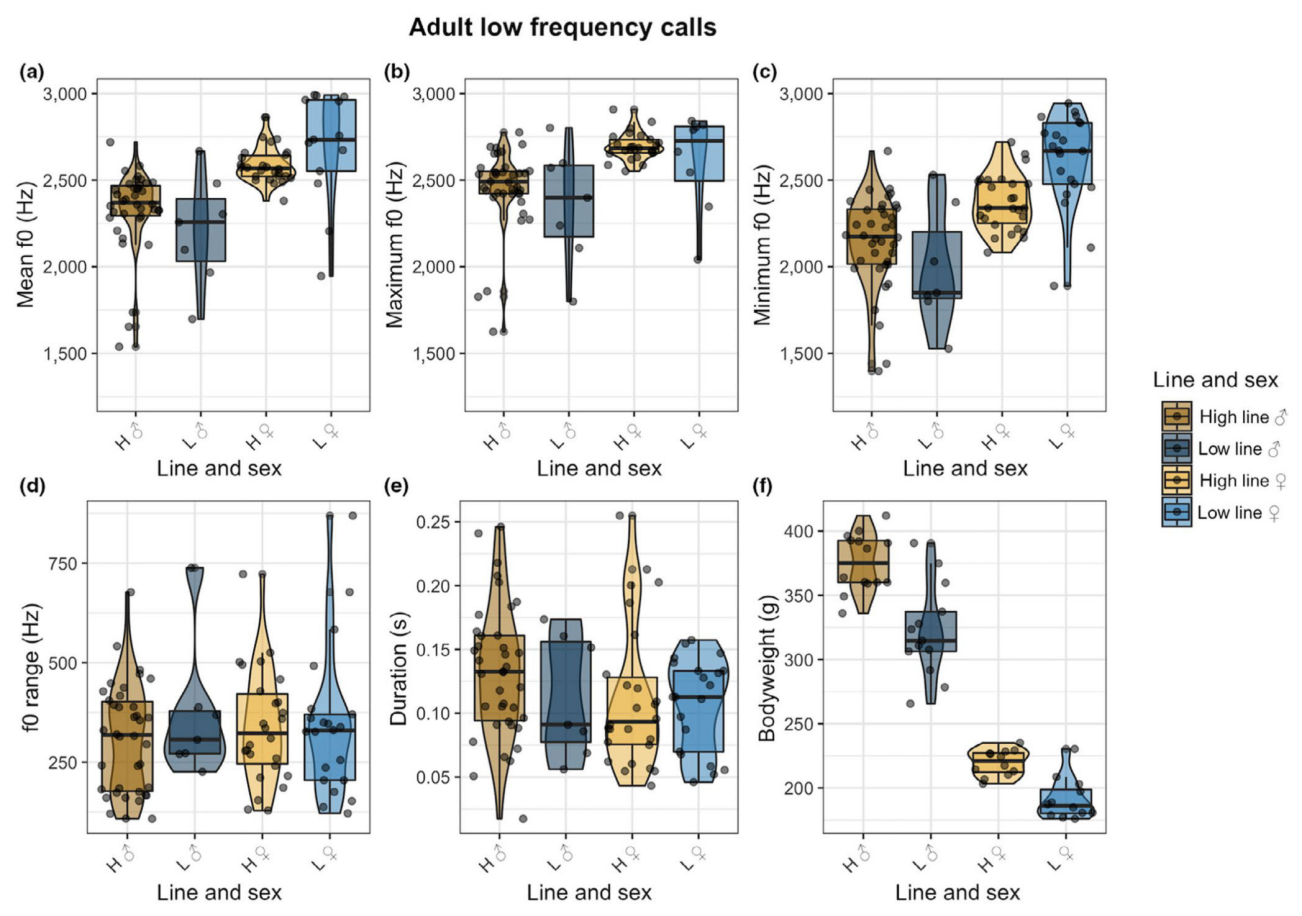
Adult audible call acoustic measurements. The different lines are indicated by letters (H = high line, L = low line) and sex is indicated by symbols (♂ = male, ♀ = female). (a) Mean f0 of adult audible calls plotted over the line and sex combinations. (b) Maximum f0 of adult audible calls plotted over the line and sex combinations. (c) Minimum f0 of adult audible calls plotted over the line and sex combinations. (d) f0 range of adult audible calls plotted over the line and sex combinations. (e) Call duration of adult audible calls over the line and sex combination. (f) Bodyweight in grams over the line and sex combination [Colour figure can be viewed at wileyonlinelibrary.com]

Finally, we come to low-frequency (audible) vocalizations. Unlike the models for adult USVs, the full models for audible vocalizations with the fixed factors line combination, bodyweight, and their interaction were a better fit than the null model for both $\bar{x}$ mean f0 (Pr(>Chisq) = 0.01803; Figure 5a; $\bar{x}\pm SD:\;\text{high}\;$ ♂: 2,450 ± 235, low ♂:2,359 ± 339, high ♀:2,710 ± 108, low ♀:2,962 ± 335) and maximum f0 (Pr(>Chisq) = 0.01864; Figure 5b; $\bar{x}\pm SD:\;\text{high}\;$ ♂: 2,330 ± 239, low ♂:2,210 ± 323, high ♀: 2,584 ± 102, low ♀:2,826 ± 310), in adult audible calls. The full model for minimum f0, f0 range and call duration were not significantly better than the null model (Figure 5c,d,e). Thus, to our surprise, selection on pup USV calls led to complex changes in adult audible calls, but not in adult USVs. Low line females produced the highest mean and maximum f0, and low line males produced both the lowest mean and maximum f0; high line males had a higher average f0 compared with the low line males.

Again (since the same individuals provided data), the full model for bodyweight was significantly better than the null model (Pr(>Chisq) < 2.2e-16; Figure 5f). However, body size was not consistently related to f0. Although the males of the high line were heavier than their low line counterparts, they produced on average a higher mean and maximum fundamental frequency. This was not true for the high line females, although they too were heavier than their low line counterparts.

## 4 Discussion

Our results indicate an unexpected pattern of changes in the low-frequency vocalizations of adult rats after selection on the USVs of rat pups. Despite our hypothesis that selection for pup USV rate should impact adult USV production without affecting the lower frequency calls, we found the opposite to be true. While adult USV call metrics did not differ, we found low-frequency (human-audible) calls to be affected by the pup USV selection procedure. Male adult rats that were bred to produce many USVs as pups (high line) had a higher fundamental frequency in audible calls than low line males. The opposite was true for female adult rats; high line females produced audible calls lower in f0 than the low line females. Apart from the findings in the adult animals, we also found interesting changes in the pup USVs. High line pups had lower f0s than their respective low line counterparts. To sum up, we analysed acoustic parameters in calls produced by rat lines selected for high or low ultrasound production in both pups and adults of both lines. We found selection pressures on neonate acoustic output to further impact the animals’ vocal communication system across development.

Our first hypothesis predicted that adult rats produce USVs in accordance with their line-specific behaviour as pups, for example low line individuals should produce few USVs as adults and high line individuals should produce many. Contrary to our prediction, we found that adult low line males produced, on average, slightly more USVs than the high line. This difference could potentially be explained by an inherently different stress response to the tickle paradigm within the two lines. High line rats have a more anxious and depressed stress response compared with low line individuals (Brunelli, 2005; Brunelli et al., 2010; Zimmerberg et al., 2005). Taking line-specific stress responses into consideration, one might reasonably expect this precise pattern of less stressed (low line) rats vocalizing more during the human interaction (tickling).

Our second hypothesis predicted that the previously observed longer and broader-band calls (Spence et al., 2016) of high line pups would also be found in our population of pups. We did not find a broader f0 range in the high line pups, but we did find a longer call duration. Furthermore, we predicted longer and broader-band calls in adult high-line USVs, but neither USV range nor duration differed between lines in adults.

Our third hypothesis predicted that USV-focussed breeding would have little measurable impact on low-frequency (audible) calls. Again, our data did not support this hypothesis. While rat line was irrelevant in predicting mean f0 of adult USV calls, rat line was highly important in predicting the data distribution of adult audible f0 call parameters.

All of our results are related to different measurements of fundamental frequency, suggesting a potential link between line-specific stress responses and its possible influence on f0 measurements (Briefer, 2012). So far, three independent rat breeding programs (Brunelli, 2005; Burgdorf, Panksepp, Brudzynski, Kroes, & Moskal, 2005; Frank et al., 2006), with different selection procedures, have linked selection on pup USV production with differences in adult anxiety. This may suggest that differences found in the acoustic parameters measured here were due to line-specific stress responses impacting f0 measurements. Schehka and Zimmermann (Schehka & Zimmermann, 2009) found an increased f0 to be an indication of emotional arousal, which again directly links to stress coping mechanisms (cf., Briefer, 2012). Consistent with this idea, we found that high line males, which are more responsive to stress and more easily aroused than their low line counterparts, produced higher frequencies than males of the low line. But the opposite is true for the females: high line females produced on average lower mean f0s in their audible calls than did low line females.

We argue that this finding, and the fact that adult rats of both lines produced 50kHz USVs (indicators of positive affective states) that did not differ in their call metrics, leads to the conclusion that acoustic line differences are not simply a reflection of different stress coping. Rather we suggest, since animals of both lines playfully engaged in social interaction with the human experimenter, the audible calls produced in this context do not reflect differing stress levels. If correct, this argument suggests that the different frequencies of audible calls may be a product of sex-dependent laryngeal differences (e.g. larynx size or mineralization, or vocal fold length).

Another peculiar finding was the distinct difference of f0 measurements in pup USVs. We found pup males to produce lower f0s than females of the same line, despite no significant difference in bodyweight. Aside from this sex dimorphism in pup USV f0, we also found high line individuals to produce lower f0s than their respective low line counterparts. Bowers and colleagues (Bowers, Perez-Pouchoulen, Edwards, & McCarthy, 2013) reported lower USV f0 in male pups and a higher number of USVs produced during maternal separation. While we did not find sex-specific differences in the number of pup USVs produced, we did find males of both lines to produce lower f0s compared with their respective lines’ females. Bower’s and colleagues’ findings help explain the sex dimorphism in pups USV f0, but not this line-specific difference. Male high line pups produced a lower f0 than low line males; the same pattern applied to females.

Our results only allow us to speculate whether these line-specific, selection-induced acoustic differences occur due to neuro-behavioural or physiological changes; they might also be found in their interplay. The multiple effects of artificial selection on one specific type of vocal output on other vocalizations (or behaviours) that we document here in rats have interesting potential implications in the context of vocal changes in domestication. Some domestic animals show somewhat different acoustic behaviours than their wild counterparts. For example, adult dogs bark more than wolves (Feddersen-Petersen, 2000) and adult domestic cats meow, but in wildcats this vocal type is common only juveniles. Physiologically, adults in both sub-species are able to produce these vocalizations, but nevertheless they produce them in different proportions, and the reasons for these vocal differences remain unclear.

More generally, our results suggest that selection on a particular vocalization type, at a particular developmental stage, may have surprising and unpredictable effects on other vocalization types and/or at other developmental stages. This has interesting implications for trade-offs in the evolution of communication (cf. Dunn et al., 2015), suggesting that (as in the evolution of body form) selection on a single trait may have a host of unselected by-product effects on other traits (Kozłowski & Weiner, 1997). The example we have uncovered in the current study is experimentally tractable, insofar as the specific selection history of the animals is known, and the physiological basis for the production of vocalizations is well-understood. The results of further investigations in our example will have implications for the evolution of communication in other species, where selection history (and perhaps details of vocal production) is not as well understood.

In summary, artificially selected line-specific differences found in early developmental stages were lost in later developmental stages, but had unpredicted effects on the acoustics of different vocalization types. The selection pressure, focused entirely on the number of USVs produced by pups, influenced the production of adult audible calls (which are based on a different acoustic production mechanism), but did not affect adult USV calls. Our results indicate a surprising form of pleiotropy that violated most of our a priori predictions. Our evaluation of body size effects demonstrates that this is not a simple result of body size changes, and also shows that vocal production characteristics can readily change under selection, independent of changes in body size. Despite difference in temperament between the two lines, we do not think that these results affect different anxiety or arousal levels either, though this should be controlled for in future research. This leaves changes in vocal anatomy between the lines as the most likely explanation of our acoustic results. Anatomical investigations into possible changes in larynx morphology are in progress to evaluate and investigate the precise nature of these effects, and thus to better understand the effects that selection for acoustic output may have on vocal organs.
